# Doppler evaluation of maternal vessels in normal gestation and threatened abortion in canines

**DOI:** 10.1186/s13620-020-00169-9

**Published:** 2020-08-01

**Authors:** Sandeep Mahadeo Gaikwad, Sarita Ulhas Gulavane, Umesh Balkrishna Kumbhar, Raju Ramrao Shelar, Ravindra Jayram Chaudhari, Ruth Ann Ribeiro

**Affiliations:** Department of Animal Reproduction Gynaecology and Obstetrics, Mumbai Veterinary College, Mumbai, Maharashtra 400012 India

**Keywords:** Uteroplacental arteries, Umbilical arteries, Systolic peak velocity, End diastolic velocity, Pulsatility index, Resistivity index

## Abstract

**Background:**

Ultrasonographic monitoring of the pregnant bitch is an established routine in many veterinary clinics. In order to better assess foetal health and avoid pregnancy losses, Doppler ultrasonographic evaluation of the pregnant uterus is the need of the day. Investigations on the dynamics of maternal and foetal blood flow using Doppler ultrasound have been conducted in multiple species recently and it is invisaged that it would be a reliable diagnostic tool in future in monitoring pregnancy. The present study was designed to compare Doppler parameters systolic peak velocity (SPV), end diastolic velocity (EDV), pulsatility index (PI) and resistivity index (RI) of the uteroplacental (UPA) and umbilical arteries (UA) measured during 40 to 50 and 51 to 60 days in normal gestation and threatened abortion in canines.

**Results:**

In total 40 pregnant bitches with known history of breeding; irrespective of age and parity belonging to different breeds were classified into normal gestation (NG) and threatened abortion (TA). Bitches with the history of greenish black discharge or expulsion of one foetus were included in the abortion group and bitches with no such symptoms were included in the normal gestation group. End diastolic velocity of uteroplacental vessels decreased while PI and RI increased significantly with decrease in body weight in threatened abortion cases during 40 to 50 and 51 to 60 days of gestation in canines. Systolic peak velocity and EDV of umbilical arteries increased while PI decreased significantly with decrease in body weight during 40 to 50 days of gestation in canine threatened abortions.

**Conclusions:**

Doppler evaluation of uteroplacental and umbilical arteries is recommended as a diagnostic tool to monitor high risk pregnancy during 40 to 50 and 51 to 60 days of gestation in canines.

## Background

Ultrasonographic monitoring of the pregnant bitch is an established practice in many veterinary clinics. Imaging of the developing foetus, various landmarks in foetal ageing, foetal heart rate monitoring etc. have been standardised over the past decade. Obstetric Doppler ultrasonography gives the gynaecologist a reliable opportunity to review foeto-maternal hemodynamics by investigating vessels like the umbilical artery and vein, uteroplacental arteries, foetal thoracic aorta, foetal caudal vena cava and foetal cerebral artery [1, 2]. Investigations on the dynamics of maternal and foetal blood flow using Doppler ultrasound have been conducted in different species recently and it is thought that it would be a reliable diagnostic tool in future [3–6]. Resistance indices of uteroplacental and umbilical arteries, foetal aorta and foetal common carotid arteries progressively decrease throughout normal canine gestation [7–10] implying an appropriate perfusion of the placenta and foetal viscera.

In canine pregnancy, the application of Doppler ultrasonography can be explored in various areas like: timely intervention for threatened abortions, deciding the time for caesarean section in prolonged pregnancies or protracted dystokias etc. The detection of the umbilical cord is possible via ultrasonography after the days 40–46 of pregnancy in canine [11].

Abnormal vascular placental development in foetal and/or maternal compartments may be an indicator of intrauterine growth restriction, foetal distress and early pregnancy failure in humans [12, 13]. An experimental canine model of abnormal gestation suggested that resistive index of the uterine artery could also be a predictor of compromised pregnancy and impending abortion in this species [14]. A reduction is heart rate has been the cardinal sign of foetal distress and impending abortion in canines. However, these abnormalities occurred before heart rate decreased, suggesting that Doppler could be an early indicator of compromised pregnancies and obstetrical diseases. However, minimal information is available on spontaneous cases of canine pathological gestation. Though Doppler ultrasonography alone is not sufficient for the evaluation of foetal well-being, this technique enables the gynaecologist to diagnose foetal distress earlier than other tests [15]. It was hypothesised that estimating uteroplacental and umblical blood flow parameters utilising Doppler ultrasound technology would result in early detection of impending abortion and further help in monitoring the outcome of the treatment in canines.

## Methods

Clinically healthy pregnant bitches, irrespective of age, weight, parity and breed with known history of breeding were selected in this study. The bitches belonged to different breeds with body weight ranging from 3 to 55 kg. The experimental animals were owned by pet owners in and around Mumbai city. A total of 40 bitches were included and classified into NG (*n* = 20) and TA (*n =* 20). They were further divided into four groups according to maternal body weight; each comprising of 10 bitches. (Group I-NG, ≥ 25 kg BW; Group II- NG, < 25 kg BW; Group III- TA, ≥ 25 kg BW and Group IV- TA < 25 kg BW). The bitches weighing exactly 25 kg were included in the above 25 kg group. Bitches with a history of greenish black discharge and /or expulsion of one foetus were included in the threatened abortion group [16]. Bithces with no such symptoms were included in normal gestation group.

**Fig. 1:**
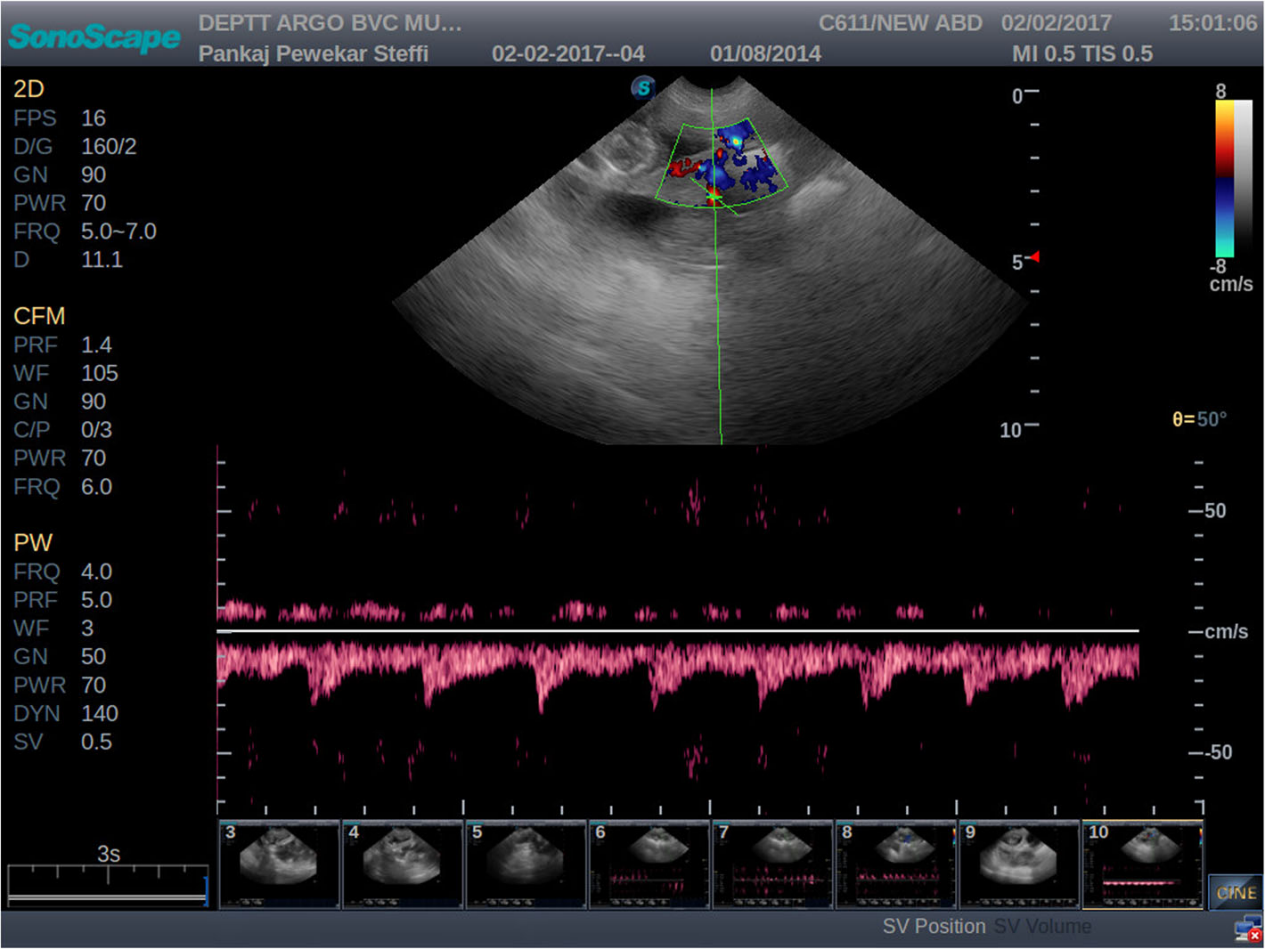
Pulsed wave Doppler image of uteroplacental arteries during 40 to 50 days of gestation in threatened abortion cases. The wave form of the uteroplacental arteries in the threatened abortion cases was quite different than the normal gestation. It was observed that during 40–50 days a systolic peak was followed by full diastole, with broadening and progressive flattening of the diastolic upstroke. This culminated in a monophasic wave with little oscillation during diastole. This wave morphology might suggest vascular pathology due to the placentitis

**Fig. 2:**
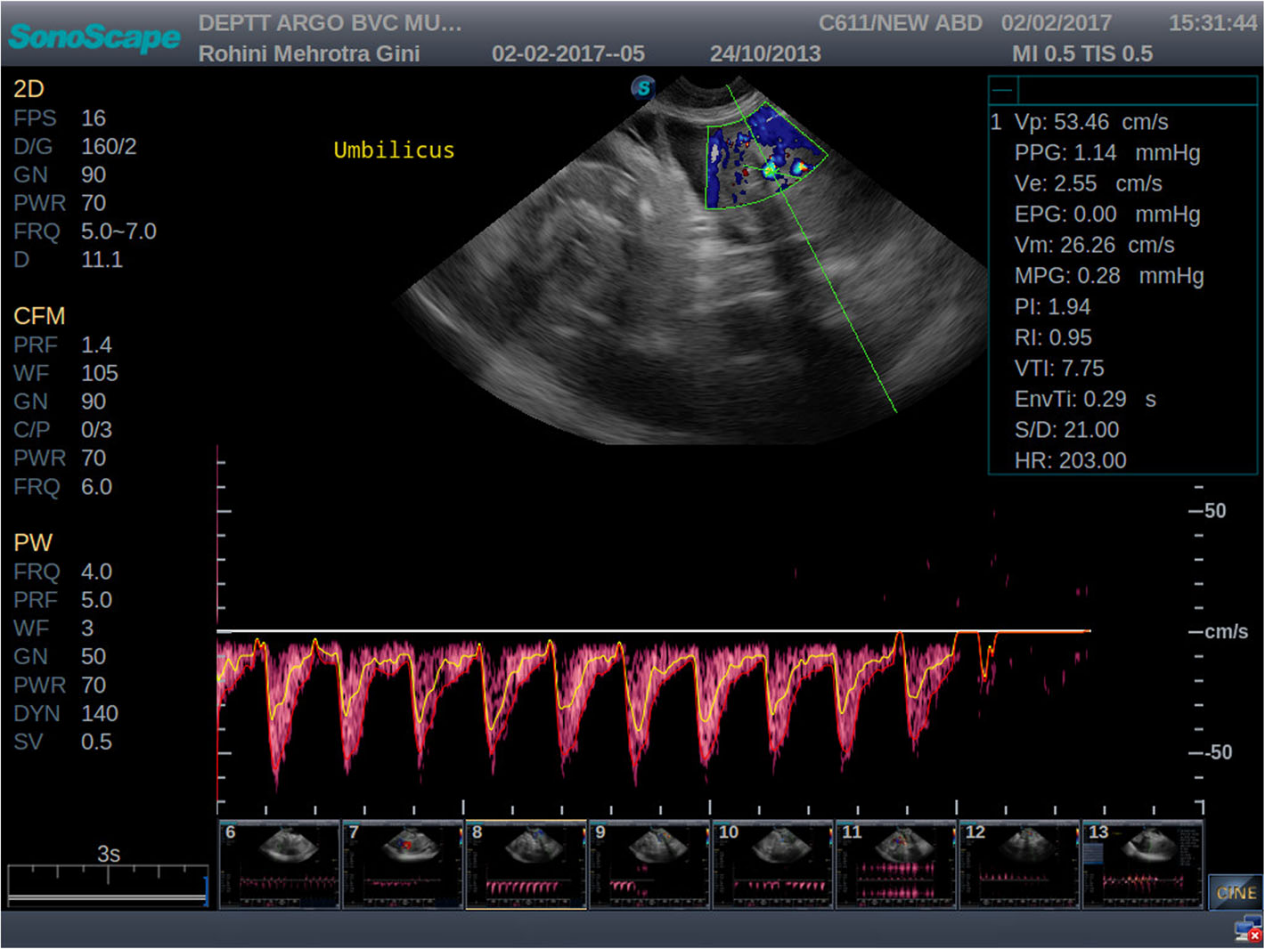
Colour Doppler and pulsed wave image of umbilical arteries during 40 to 50 days of gestation. Umbilical arteries with only systolic blood flow on 42 days of gestation in canines

All the experimental bitches were subjected to ultrasound examination during 40 to 50 and 51 to 60 days of gestation. Colour and pulse wave Doppler ultrasound examination of the uteroplacental vessels and umbilical arteries was performed. Compared to velocimetry of arcuate arteries, Doppler sonography of the uterine arteries in human permits a more general evaluation of uterine perfusion. In humans the vaginal route is preferred to trans-abdominal scanning as it has shown to eliminate errors due to its proximity to the uterine vessels [17]. This approach however, is not possible in bitches without sedation. Also, it is difficult to locate the uterine arteries trans-abdominally in advanced gestation [17]. Hence, in the present study, the uteroplacental arteries were studied rather than the uterine arteries. Utero-placental arteries were scanned either adjacent to the gestational sac or between two gestational sacs. Blood flow was detected by imaging the area mentioned and applying colour Doppler mode of the ultrasonography machine. Once the blood flow was detected, a pulsed-wave Doppler was performed to obtain the waveform of the uteroplacental arteries [Fig. 1 and Fig. 2]. The Doppler waveform obtained on pulsed wave evaluation was frozen and the values for SPV, EDV were obtained by manually tracing three consecutive waveforms. The PI and RI were obtained automatically from the machine itself.

The umbilicus was identified free floating in the amniotic fluid; connecting the allantois to the foetus at the abdomen. The umbilical artery was identified with colour flow mapping in the umbilical cord near the placental surface. The arteries and vein in the mid-cord site of the free-floating umbilical cord were examined. The Doppler gate was placed in the middle of the pulsating vessel to obtain the waves. Qualitative waveform analysis was done by noting the typical appearance of the waveform. In particular, the vessels were visualized by colour Doppler and then a PW sample volume was placed exactly in the centre of the colour-coded blood flow and the waveforms with at least three consecutive cardiac cycles were recorded. The SPV, EDV, RI, and PI values were obtained and the vessel was evaluated qualitatively. Data regarding the blood flow parameters of the uteroplacental arteries and umbilical arteries of the bitches during 40 to 50 and 51 to 60 days of gestation in all groups was statistically analysed using t-test. This study did not require official or institutional ethical approval; however the project was formally approved by IEC for Veterinary Clinical Research at Bombay Veterinary College, Mumbai and Board of Studies, Maharashtra Animal and Fishery Sciences University Nagpur. The animals were handled according to high ethical standards and national legislation.

## Results

### Doppler evaluation of uteroplacental arteries in normal gestation groups (I and II) (Tables 1 and 2)

The results of the analysis revealed that the blood flow parameters viz. SPV, EDV, PI, and RI in group I and II did not differ significantly (*p>* 0.05) during 40 to 50 and 51 to 60 days of gestation. It indicates that in normal gestation body weight has no significant effect on blood flow parameters of uteroplacental arteries during 40 to 50 and 51 to 60 days of normal gestation in canines. The SPV of uteroplacental arteries increased gradually while EDV increased significantly from 40 to 50 to 51 to 60 days of gestation. The blood flow indices viz. PI and RI gradually decreased with advancing gestation in the present study. There are no references to compare values and trends in threatened abortion cases in canines, however, these values and trends are compared with normal gestation.

**Table 1:** Blood flow parameters of the uteroplacental arteries in group I and II during 40 to 50 days

Sr. No	Parameter	40 to 50 days		‘t’ Cal	‘t’ Table
		Group I (*n* = 10)	Group II (*n* = 10)		
1	SPV (cm/sec)	31.57 ± 4.56	31.42 ± 4.78	0.07	2.10
2	EDV (cm/sec)	14.02 ± 5.70	15.49 ± 3.28	0.61	2.30
3	PI	00.89 ± 0.43	00.90 ± 0.45	0.02	2.10
4	RI	00.57 ± 0.16	00.57 ± 0.16	0.02

**Table 2:** Blood flow parameters of the uteroplacental arteries in group I and II during 51 to 60 days

Sr. No	Parameter	51 to 60 days		‘t’ Cal	‘t’ Table
		Group I (*n* = 10)	Group II (*n* = 10)		
1	SPV (cm/sec)	34.49 ± 4.67	33.48 ± 5.13	0.45	2.10
2	EDV (cm/sec)	18.95 ± 3.58	18.79 ± 3.43	0.10	
3	PI	00.64 ± 0.11	00.59 ± 0.09	1.05	
4	RI	00.45 ± 0.07	00.44 ± 0.07	0.44	2.26

### Doppler evaluation of uteroplacental arteries in threatened abortion groups (III and IV) (Tables 3 and 4)

End diastolic velocity in group IV was significantly lower than group III, while, PI and RI of uteroplacental arteries were significantly higher in group IV than III during 40 to 50 and 51 to 60 days of gestation. It indicates that in threatened abortion cases, the body weight of the female has a significant effect on EDV, PI and RI of the uteroplacental arteries in canines during 40 to 50 days of gestation. In threatened abortion cases, body weight had no significant effect on SPV values of uteroplacental arteries in canines.

**Table 3:** Blood flow parameters of the uteroplacental arteries in group III and IV during 40 to 50 days

Sr. No	Parameter	40 to 50 days		‘t’ Cal	‘t’ Table
		Group III (***n*** = 10)	Group IV (***n*** = 10)		
1	SPV (cm/sec)	30.75 ± 4.32	30.57 ± 4.98	0.08	2.101
2	EDV (cm/sec)	07.89 ± 1.86^a^	06.00 ± 1.42^b^	2.54	
3	PI	01.06 ± 0.10^a^	01.28 ± 0.16^b^	3.63	
4	RI	00.74 ± 0.05^a^	00.80 ± 0.03^b^	3.05	

[Different superscripts within same row denote significant differences (*p* < 0.05).]

**Table 4:** Blood flow parameters of the uteroplacental arteries in group III and IV during 51 to 60 days

Sr. No	Parameter	51 to 60 days		‘t’ Cal	‘t’ Table
		Group III (***n*** = 07)	Group IV (***n*** = 08)		
1	SPV (cm/sec)	35.25 ± 3.82	35.83 ± 15.19	0.10	2.37
2	EDV (cm/sec)	10.43 ± 1.84^a^	07.55 ± 1.38^b^	2.54	2.16
3	PI	00.96 ± 0.09^a^	01.71 ± 0.27^b^	6.88	
4	RI	00.70 ± 0.07^a^	00.80 ± 0.02^b^	3.84	2.16

[Different superscripts within row denote significant differences (*p* < 0.05).]

### Doppler evaluation of umbilical arteries in normal gestation groups (I and II) (Tables 5 and 6)

**Fig. 3:**
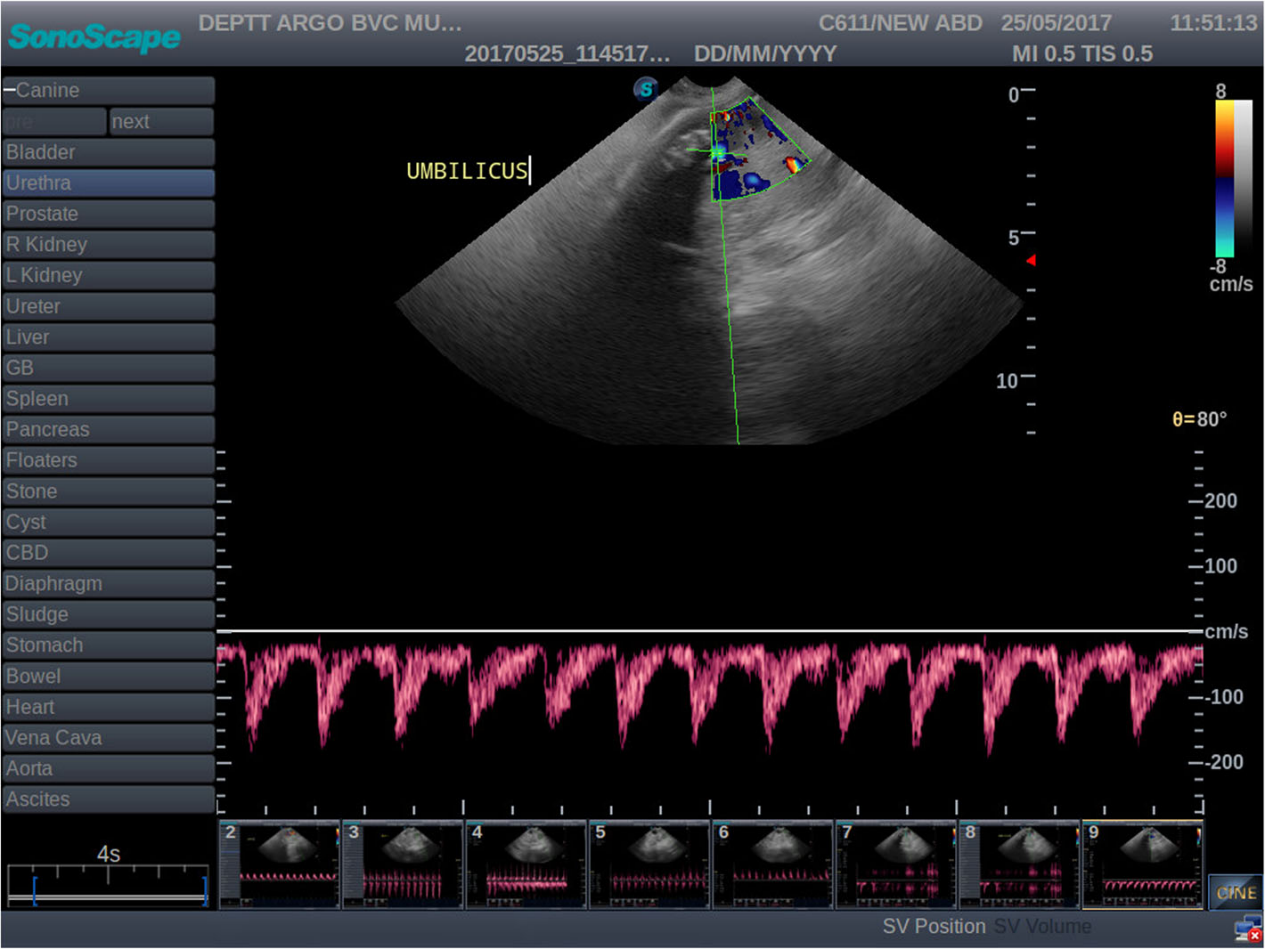
Color Doppler and pulsed wave image of umbilical arteries during 51 to 60 days of gestation. Umbilical arteries with systolic blood flow with diastolic waveform

Amongst the ten bitches, two bitches were presented on day 41 and 42 of gestation where umbilical arteries showed only systolic blood flow (Fig. 3). In the remaining eight bitches which were presented beyond 42 days of gestation, diastolic waveform was detected in the umbilical artery.

**Table 5:** Blood flow parameters of the umbilical arteries in group I and II during 40 to 50 days

Sr. No	Parameter	40 to 50 days		‘t’ Cal	‘t’ Table
		Group I (*n* = 10)	Group II (*n* = 10)		
1	SPV (cm/sec)	29.89 ± 6.33	35.38 ± 10.94	1.37	2.101
2	EDV (cm/sec)	01.55 ± 1.50^a^	05.21 ± 1.17^b^	6.09	
3	PI	01.58 ± 0.13^a^	01.31 ± 0.14^b^	4.64	
4	RI	00.94 ± 0.05^a^	00.84 ± 0.05^b^	4.38	

[Different superscripts within same row denote significant differences (*p* < 0.05).]

**Table 6:** Blood flow parameters of the umbilical arteries in group I and II during 51 to 60 days

Sr. No	Parameter	51 to 60 days		‘t’ Cal	‘t’ Table
		Group I (n = 10)	Group II (*n* = 10)		
1	SPV (cm/sec)	38.94 ± 10.14	41.80 ± 9.35	0.65	2.101
2	EDV (cm/sec)	08.91 ± 2.37	08.54 ± 1.35	0.42	
3	PI	01.31 ± 0.21	01.35 ± 0.24	0.34	
4	RI	00.76 ± 0.06	00.79 ± 0.05	0.96

The blood flow parameters viz. EDV, PI and RI during 40 to 50 days of gestation differed significantly (*p <* 0.05) in group I and II while no significant difference (*p*> 0.05) was observed in SPV values of umbilical arteries. End diastolic velocity was significantly higher in group II, while, PI and RI value was significantly lower in group II than I during 40 to 50 days of gestation. However, the blood flow parameters viz. SPV, EDV, PI and RI in group I and II did not differ significantly (*p>* 0.05) during 51 to 60 days of gestation. It indicates that in normal gestation, body weight has a negative and significant effect on EDV values while it has positive and significant effect on values of PI and RI of umbilical arteries during 40 to 50 days of gestation in canines.

The blood flow velocities of the umbilical arteries increased with advancement of gestation. There was a significant increase in SPV and EDV of umbilical arteries during 51 to 60 days of gestation. The blood flow indices decreased significantly with advancement of gestation from 40 to 50 to 51 to 60 days. The indices were positively correlated with each other (*p* < 0.05) and negatively with the EVD (*p <* 0.05) during 40 to 50 days while during 51 to 60 days there was non-significant (*p* > 0.05) negative correlation with EDV.

### Doppler evaluation of umbilical arteries in threatened abortion groups (III and IV) (Tables 7 and 8)

Restivity index of umbilical arteries in group III and IV did not differ significantly during 40 to 50 days of gestation, while, SPV, EDV and PI differed significantly (*p* < 0.05). It indicates that in threatened abortion cases, body weight has no significant effect on RI of umbilical arteries in canines. However, SPV and EDV for umbilical arteries in group IV was significantly higher (*p* < 0.05) than group III, while, PI of umbilical arteries was significantly lower (*p <* 0.05) in group IV than III indicating a significant effect of body weight on SPV, EDV and PI values of the umbilical arteries in canine threatened abortions during 40 to 50 days of gestation. During 51 to 60 days of gestation, SPV, EDV and RI did not differ significantly while PI was significantly higher in group III than group IV during 51 to 60 days of gestation. It indicates that in threatened abortion cases, body weight of the female has a significant effect on PI values of umbilical arteries, during 51 to 60 days of gestation, in canines.

**Table 7:** Blood flow parameters of the umbilical arteries in group III and IV during 40 to 50 days

Sr. No	Parameter	40 to 50 days		‘t’ Cal	‘t’ Table
		Group III (***n*** = 10)	Group IV (***n*** = 10)		
1	SPV (cm/sec)	31.54 ± 5.78^a^	37.95 ± 6.19^b^	2.39	2.10
2	EDV (cm/sec)	03.69 ± 0.75^a^	04.58 ± 0.38^b^	3.21	2.11
3	PI	01.56 ± 0.13^a^	01.30 ± 0.23^b^	3.08	2.10
4	RI	00.89 ± 0.01	00.89 ± 0.04	0.28	2.26

[Different superscripts within same row denote significant differences (*p* < 0.05).]

**Table 8:** Blood flow parameters of the umbilical arteries in group III and IV during 51 to 60 days

Sr. No	Parameter	51 to 60 days		‘t’ Cal	‘t’ Table
		Group III (***n*** = 07)	Group IV (***n*** = 08)		
1	SPV (cm/sec)	41.22 ± 7.33	43.66 ± 4.67	0.78	2.16
2	EDV (cm/sec)	08.98 ± 2.45	09.77 ± 1.07	0.85	
3	PI	01.81 ± 0.36^a^	01.07 ± 0.02^b^	5.86	
4	RI	00.78 ± 0.04	00.77 ± 0.01	0.49	

[Similar superscripts within same row denote non-significant differences (*p*>0.05).]

The SPV and EDV of umbilical arteries in threatened abortion groups increased significantly while RI decreased significantly and the PI values increased numerically with advancement of gestation. The indices were negatively but non-significantly correlated with each other (*p* < 0.05) and also with the EDV (*p <* 0.05). Higher PI (> 1.51) and RI (≥ 0.90) values were observed in three bitches from group III and two from group IV which aborted before their scanning during 51 to 60 days of gestation. The RI of the umbilical artery was observed to be greater due to a minor augmentation of EDV.

### Overall comparison of blood flow parameters of uteroplacental arteries in normal gestation and threatened abortion groups (Tables 9 and 10)

**Table 9:** Blood flow parameters of the uteroplacental arteries in normal gestation and threatened abortion groups during 40 to 50 days

Sr. No	Parameter	40 to 50 days		‘t’ Cal	‘t’ Table
		Normal gestation (***n*** = 20)	Threatened abortion (***n*** = 20)		
1	SPV (cm/sec)	31.49 ± 4.55	30.66 ± 4.53	0.57	2.02
2	EDV (cm/sec)	15.54 ± 3.07^a^	06.95 ± 1.87^b^	10.52	
3	PI	00.89 ± 0.42^a^	01.17 ± 0.17^b^	2.65	
4	RI	00.56 ± 0.15^a^	00.77 ± 0.05^b^	5.49	

[Different superscripts within row denote significant differences (*p* < 0.05).]

**Table 10:** Blood flow parameters of the uteroplacental arteries in normal gestation and threatened abortion groups during 51 to 60 days

Sr. No	Parameter	51 to 60 days		‘t’ Cal	‘t’ Table
		Normal gestation (***n*** = 20)	Threatened abortion (***n*** = 15)		
1	SPV (cm/sec)	33.98 ± 4.80	37.44 ± 5.60	1.96	2.03
2	EDV (cm/sec)	18.87 ± 3.40^a^	08.89 ± 3.32^b^	9.74	
3	PI	00.61 ± 0.10^a^	01.36 ± 0.43^b^	7.40	
4	RI	00.44 ± 0.06^a^	00.75 ± 0.07^b^	12.68	

[Different superscripts within row denote significant difference (*p* < 0.05).]

### Overall comparison of the blood flow parameters of umbilical arteries in normal gestation and threatened abortion groups (Tables 11 and 12)

**Table 11:** Blood flow parameters of the umbilical arteries in normal gestation and threatened abortion groups during 40 to 50 days

Sr. No	Parameter	40 to 50 days		‘t’ Cal	‘t’ Table
		Normal gestation (***n*** = 20)	Threatened abortion (***n*** = 20)		
1	SPV (cm/sec)	32.63 ± 9.14	37.74 ± 6.69	0.83	2.02
2	EDV (cm/sec)	03.37 ± 2.28	04.11 ± 0.74	1.33	
3	PI	01.44 ± 0.18	01.43 ± 0.22	0.23	
4	RI	00.89 ± 0.07	00.88 ± 0.03	0.53	

**Table 12:** Blood flow parameters of the umbilical arteries in normal gestation and threatened abortion groups during 51 to 60 days

Sr. No	Parameter	51 to 60 days		‘t’ Cal	‘t’ Table
		Normal gestation (***n*** = 20)	Threatened abortion (***n*** = 15)		
1	SPV (cm/sec)	40.37 ± 9.60	42.52 ± 5.96	0.76	2.03
2	EDV (cm/sec)	08.72 ± 1.88	09.39 ± 1.77	1.07	
3	PI	01.33 ± 0.22	01.42 ± 0.45	0.74	
4	RI	00.77 ± 0.05	00.77 ± 0.02	0.26	

## Discussion

Maternal bodyweight in canines has no significant effect on blood flow parameters of uteroplacental arteries during 40 to 50 and 51 to 60 days of normal gestation. In the present study, During 40 to 50 days of gestation, the SPV values of uteroplacental arteries in normal gestation groups and threatened abortion groups did not differ significantly, while EDV, PI and RI differed significantly. End diastolic velocity was significantly lower, while PI and RI values were significantly higher in threatened abortion groups than normal gestation groups. The blood flow pattern observed in the uteroplacental arteries of canines is typical of low resistance arteries. This might be due the functional modification that facilitates a large increase in placental perfusion during pregnancy which is similar to what is observed in pregnant felines and women despite a different type of placentation. The wave form of the uteroplacental arteries in threatened abortion cases was quite different than that of normal gestation. It was observed that during 40 to 50 days a systolic peak was followed by full diastole, with broadening and progressive flattening of the diastolic upstroke. This culminated in a monophasic wave with little oscillation during diastole. This wave morphology might suggest vascular pathology. A monophasic wave with little oscillation during diastole is suggestive of vascular stenosis of uteroplacental artereis in canines [18]. When data regarding EDV of uteroplacental arteries in normal gestation and threatened groups was pooled together it was observed that, EDV of uteroplacental arteries ranged between 11.77 to 19.49 during 40 to 50 days of gestation, while, in threatened abortion groups it ranged between 3.89 to 10.50 which was significantly lower. So it may be concluded that during 40 to 50 days of gestation in canines if the EDV of uteroplacental arteries is above 11.77, such pregnancy can be considered as normal. However, if the EDV is below 10.50, it may be considered as threatened abortion and should be monitored carefully. During 51 to 60 days of gestation, the SPV values of uteroplacental arteries, in normal gestation groups and threatened abortion groups did not differ significantly, while EDV, PI and RI differed significantly in the present study. The end diastolic velocity was significantly lower, while PI and RI values were significantly higher in threatened abortion groups than normal gestation groups. PI of the spiral arteries in threatened abortion was significantly higher than normal pregnancy in women during the first trimester [19]. However, women with threatened abortion showed a significantly lower RI of the uterine artery, in the first trimester, compared to those with normal pregnancies [20].

When data regarding RI of uteroplacental arteries in normal gestation and threatened groups was pooled together it was observed that, RI of uteroplacental arteries ranged between 0.42 to 0.59 and 0.29 to 0.51 during 40 to 50 and 51 to 60 days of gestation, respectively, while, in threatened abortion groups it ranged between 0.63 to 0.86 and 0.58 to 0.85; which was significantly higher. So it may be concluded that during 40 to 50 days of gestation in canines if the RI of uteroplacental arteries is below 0.59 after EDV is measurable, such pregnancy may be considered as normal. However, if the RI is above 0.63 after EDV is measurable, it may be considered as threatened abortion and should be monitored carefully. The wave form of the uteroplacental arteries in the threatened abortion cases during 40–50 days shows a systolic peak was followed by full diastole, with broadening and progressive flattening of the diastolic upstroke. This culminated in a monophasic wave with little oscillation during diastole.

In the present study, the umbilical artery waveform was only systolic up to 42 days of gestation; later a diastolic peak appeared. The observations of the present study were in agreement with the observations of Nautrup (1998) and Di Salvo et al. (2006) for canines and Scotti et al. (2008), Pereira et al. (2012) for felines in the given time periods. The umbilical vein is flat during all weeks of pregnancy. During initial ultrasound examination, the absence of the diastolic flow in the umbilical artery and the simultaneous presence of maximum PI and RI values demonstrate the high blood flow resistance present in the canine placenta. With advancement of gestation, the appearance of the diastolic peak and the progressive development of the foetal/placental circulation are responsible for the RI drop. These findings also agree with studies in humans, where this artery has shown to be useful for identification of foetuses that are at risk. It has also been suggested that an absent or reversed EDV in the umbilical arteries is associated with a greater rate of perinatal mortality and serious morbidity among survivors [21]. However, in the present study, diastolic flow of the umbilical artery was detected after day 42 of gestation and there was not an absence or reversal after this point in gestation in any of the bitches in this group. There is no literature regarding Doppler studies in threatened abortion to compare the present findings. The difference in the readings might be due to the pathological conditions of the pregnancy and the change in the blood flow pattern. During 40 to 50 days of gestation, SPV and EDV values of UA were numerically higher while, PI and RI were lower in threatened abortion groups than normal gestation. The reliability of the Doppler exam during canine pregnancy is conditioned by the operator’s experience, especially in order to obtain the same range of angle correction (0–20^0^) in all arteries examined. It is also influenced by foetal and maternal movements as well as by the need to spend a long time in carrying out the Doppler examination. However, there are no side effects for the mother, the foetus or the operator [15].

## Conclusion

The blood flow patterns observed in the uteroplacental arteries of canines in normal gestation is typical of the low resistance arteries, which might be due the functional aspect that facilitates the large increase in placental perfusion during pregnancy. The blood flow indices were positively correlated with each other (*p* < 0.001) and negatively with the EVD (*p* < 0.05) during the study period.

The wave form of the uteroplacental arteries in the threatened abortion cases during 40–50 days shows a systolic peak was followed by full diastole, with broadening and progressive flattening of the diastolic upstroke. End diastolic velocity of uteroplacental arteries decreased while PI and RI increased significantly with decrease in body weight in threatened abortion cases during 40 to 50 and 51 to 60 days of gestation in canines. During 40 to 50 days of gestation in canines if the EDV of uteroplacental arteries is above 11.77 cm /sec, such pregnancy may be considered as normal. However, if EDV is below 10.50 cm/sec, it may be considered as threatened abortion and should be monitored carefully. During 40 to 50 days of gestation in canine if the RI of uteroplacental arteries below 0.59 after EDV is measurable; such pregnancy may be considered as normal. However, if RI is above 0.63, it may be considered as threatened abortion and should be monitored carefully.

Systolic peak velocity and EDV of umbilical arteries increased while PI decreased significantly with decrease in body weight during 40 to 50 days of gestation in canine threatened abortions. End diastolic velocity and RI of umbilical arteries may be used for diagnosing and monitoring threatened abortion during 40 to 50 days of canine pregnancy.

## Data Availability

“The datasets used and/or analyzed during the current study are available from the corresponding author on reasonable request.”

## Abbreviations

NG: Normal gestation
TA: Threatened Abortion
UPA: Uteroplacental Arteries
UA: Umbilical Arteries
SPV: Systolic Peak Velocity
EDV: End Diastolic Velocity
PI: Pulsatility Index
RI: Resistivity Index
